# Incidence, Treatment, and Outcome Trends of Necrotizing Enterocolitis in Preterm Infants: A Multicenter Cohort Study

**DOI:** 10.3389/fped.2020.00188

**Published:** 2020-05-13

**Authors:** Carlos Zozaya, Inés García González, Alejandro Avila-Alvarez, Niki Oikonomopoulou, Tomás Sánchez Tamayo, Enrique Salguero, Miguel Saenz de Pipaón, Fermín García-Muñoz Rodrigo, María L. Couce

**Affiliations:** ^1^Division of Neonatology, Hospital for Sick Children, Toronto, ON, Canada; ^2^Neonatology Department, Complexo Hospitalario Universitario de Santiago de Compostela, Health Research Institute of Santiago de Compostela, A Coruña, Spain; ^3^Neonatal Unit, Department of Paediatrics, Complexo Hospitalario Universitario A Coruña, Institute for Biomedical Research A Coruña, A Coruña, Spain; ^4^Neonatology Department, Malaga Regional Hospital, Malaga Biomedical Research Institute-IBIMA, Malaga, Spain; ^5^Neonatology Department, Hospital Universitario La Paz, Hospital La Paz Institute for Health Research, Madrid, Spain; ^6^Red Samid, Maternal and Child Health and Development Research Network, Carlos III Health Institute, Madrid, Spain; ^7^Division of Neonatology, Complejo Hospitalario Universitario Insular Materno-Infantil, Las Palmas de Gran Canaria, Spain

**Keywords:** necrotizing enterocolitis, preterm infant, mortality, morbidity, trends

## Abstract

**Background:** Data regarding the incidence and mortality of necrotizing enterocolitis trends are scarce in the literature. Recently, some preventive strategies have been confirmed (probiotics) or increased (breastfeeding rate). This study aims to describe the trends of necrotizing enterocolitis incidence, treatment, and mortality over the last decade in Spain.

**Methods:** Multicenter cohort study with data from the Spanish Neonatal Network–SEN1500 database. The study period comprised from January 2005 to December 2017. Preterm infants <32 weeks of gestational age at birth without major congenital malformations were included for analysis. The main study outcomes were necrotizing enterocolitis incidence, co-morbidity (bronchopulmonary dysplasia, late-onset sepsis, cystic periventricular leukomalacia, retinopathy of prematurity, acute kidney injury), mortality, and surgical/non-surgical treatment.

**Results:** Among the 25,821 included infants, NEC incidence was 8.8% during the whole study period and remained stable when comparing 4-year subperiods. However, more cases were surgically treated (from 48.8% in 2005–2008 to 70.2% in 2015–2017, *p* < 0.001). Mortality improved from 36.7% in the 2005–2008 to 26.6% in 2015–2017 (*p* < 0.001). Breastfeeding rates improved over the studied years (24.3% to 40.5%, *p* < 0.001), while gestational age remained invariable (28.5 weeks, *p* = 0.20). Prophylactic probiotics were implemented during the study period in some units, reaching 18.6% of the patients in 2015–2017.

**Conclusions:** The incidence of necrotizing enterocolitis remained stable despite the improvement regarding protective factors frequency. Surgical treatment became more frequent over the study period, whereas mortality decreased.

## Introduction

Necrotizing enterocolitis (NEC), one of the most important causes of morbidity and mortality in preterm infants, is related to dysbiosis, severe inflammation, and ischemic necrosis of the intestinal wall (1). The most consistent risk factor is the prematurity. Indeed, 90% of the cases occur in newborns less than 32 weeks of gestation, and there is an inverse relationship between gestational age and NEC incidence (2). The incidence has remained comparatively stable over the last years, ~6–10% of the very low birthweight infants (1). Nevertheless, it is widely variable according to the literature, ranging from 1.6% in Japan to 22% in Sweden (3, 4).

Over the last decades, some preventive strategies have been implemented (probiotics) or improved (formula rate reduction): whereas no changes regarding the medical treatment have been introduced other than improvements in general neonatal intensive care. Antibiotics, bowel rest and decompression, and supportive intensive care measures remained the standard of care. Surgery in case of perforation, but also in selected cases—clinical deterioration despite medical treatment—might be also part of the treatment. Mortality rate may reach up to 20–45%, which makes NEC one of the leading causes of death in preterm infants (5, 6).

The present study aims to report the incidence, rate of associated co-morbidity, need for surgical treatment, and mortality in very low birthweight neonates included in the SEN1500 database and describe their trends throughout the years 2005–2017 to identify weak points and potential improvement areas.

## Methods

### Study Design

This is a multicenter cohort study. Data have been extracted from the Spanish Neonatal Network SEN1500 database. In this database, data about all admitted infants born <1500 g at birth are collected (7). For this study, only patients born from 24 to 31+6 weeks gestational age were selected. Patients with major congenital malformations were excluded from the analysis. The study period comprises from January 2005 to December 2017. For data analysis, it has been divided into four sub-periods: 2005–2008, 2009–2011, 2012–2014, and 2015–2017.

### Outcomes and Definitions

NEC was diagnosed in the participant centers based on a pre-specified definition (SEN1500 Manual of Operations) that adopted the Vermont Oxford Network definition (8). Accordingly, NEC was diagnosed based:

- On the findings during surgery,
- On a post-mortem study, or
- On at least one of the following clinical criteria: bilious gastric aspirate or emesis, abdominal distension, or gross or occult blood in the stool, and at least one of the following radiological findings: pneumatosis intestinalis, hepatobiliary gas, or pneumoperitoneum.

NEC was classified in the database as non-surgical/surgical (laparotomy with or without intestinal resection or peritoneal drain insertion), depending on the treatment reported.

Bronchopulmonary dysplasia (BPD) was considered as oxygen at 36 weeks postmenstrual age (moderate-severe BPD) (9). Late-onset sepsis (LONS) was considered if confirmed by blood and/or cerebrospinal fluid (CSF) culture after the third day of life. Cystic periventricular leukomalacia was diagnosed by head ultrasound and defined as multiple small cystic changes affecting the periventricular white mater. Acute kidney injury was defined as oligo-anuria and/or endogenous plasmatic creatinine > 1.5 mg/dl. Small for gestational age was considered as birth weight < 10th percentile based on the 2013 Fenton reference curves (10). Exclusive breast milk feeding at discharge was the only nutritional data available in the original database and was used as a surrogate of exclusive human milk feeding in this study.

### Statistical Analysis

Descriptive results are reported as mean ± standard deviation or percentage (*n*/*n* denominator), depending on the type of variable. Univariable analysis has been done applying either two-tailed Student *t* test or exact Fisher test as indicated. Logistic regression models adjusting for confounding factors have been built. ANOVA was used to compare means between more than two groups. Statistical analysis has been performed with Stata 13.1 statistical software (StataCorp, Texas, USA).

## Results

A total of 25,821 neonates were included in the present study analysis after 1,324 neonates were excluded because of major congenital malformations. The demographic characteristics of the included infants without NEC and with non-surgical and surgically treated NEC are shown in Table 1. Patients were distributed in the different subperiods as follows: 8,103 patients (731 with NEC) in 2005–2008, 6250 patients (554 with NEC) in 2009–2011, 6312 neonates (532 with NEC) in 2012–2014, and 5156 neonates (440 with NEC) in 2015–2017. The mean gestational age in the cohort remained unchanged over the study period: 28.5 ± 2.1 (2005–2008), 28.5 ± 2.1 (2009–2011), 28.6 ± 2.1 (2012–2014), and 28.5 ± 2.1 (2015–2017) (*p* = 0.20).

**Table 1:** Demographic characteristics of the study population.

**Study population (*n* = 25,821)**	
Sex (male)	52.3 (13,483/25,764)
Birth weight (g)	1066.75 ± 266.79
Length at birth (cm)	36.58 ± 3.42
HC at birth (cm)	28.85 ± 2.31
Small for gestational age	12.5 (3176/25,367)
Assisted reproductive technology	19.7 (4709/23,889)
Multiple gestation	34.2 (8.835/25.816)
Complete course prenatal steroids	67.2 (17,085/25,412)
Delivery (C-section)	68.7 (8094/25,820)
Apgar score <7 at 5 min	13.8 (3567/25,821)
Advanced resuscitation at birth	38.3 (9847/25,703)
Invasive mechanical ventilation	59.4 (15,287/25,723)
BPD (O2 at 36 weeks)	18 (3264/18,134)
Patent ductus arteriosus	28.7 (7210/25,164)
Late-onset sepsis	35.2 (7638/21,712)
Acute kidney injury	8.7 (2058/23,754)
GMH/IVH	28.3 (6773/23,955)
IVH grade III or PHI	10.1 (2427/23,955)
Cystic leukomalacia	2.6 (663/25,190)
Retinopathy of prematurity	24 (4878/20,305)
Necrotizing enterocolitis	8.8 (2257/25,665)
Surgical necrotizing enterocolitis	5.5 (1402/25,680)
Death	14.5 (3738/25,805)
Surfactant	57.2 (14,650/25,600)
Steroids for BPD prevention/treatment	7.4 (1887/25,690)
Inotropes	31.1 (7595/24,451)
Probiotics	14.4 (1444/10,033)
Exclusive breastfeeding at discharge	35.4 (7977/22,541)

*Data are presented as mean ± standard deviation or % (n/n of patients with valid data). BPD, bronchopulmonary dysplasia; HC, head circumference; GMH-IVH, germinal matrix hemorrhage–intraventricular hemorrhage; PHI, periventricular hemorrhagic infarction*.

### Incidence, Treatment, and Mortality Trends

**Figure 1:**
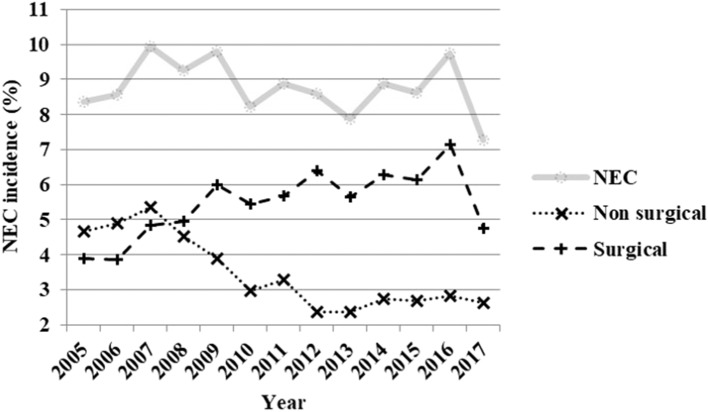
Evolution of necrotizing enterocolitis incidence—NEC (all cases), only medically treated and surgically treated cases—by year from 2005 to 2017.

**Figure 2:**
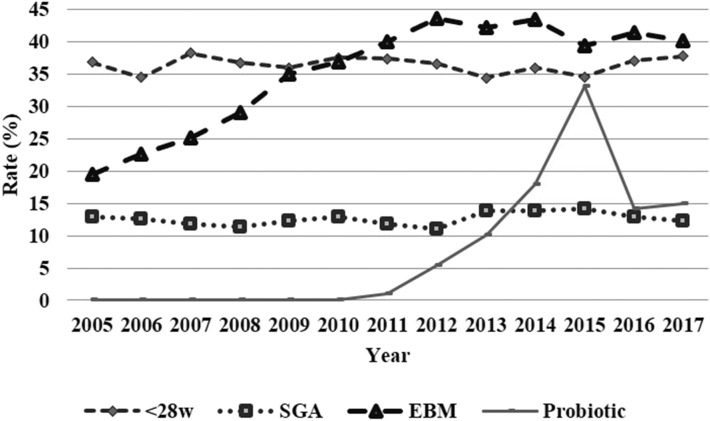
Evolution of risk and protective factors for necrotizing enterocolitis (inferior) by year from 2005 to 2017.

The average NEC incidence in the cohort over the whole study period was 8.8%. The incidence of surgically treated NEC was 5.5%, which represents 62.3% of all NEC cases. NEC incidence trends over the study period by year are depicted in Figure 1. Overall, NEC incidence remained stable: 9.1% (2005–2008), 9% (2009–2011), 8.5% (2012–2014), and 8.6% (2015–2017) (*p* = 0.521). Medically treated NEC incidence trended down: 4.9% (2005–2008), 3.4% (2009–2011), 2.49% (2012–2014), and 2.71% (2015–2017) (*p* < 0.001). On the contrary, there was a significant rise in the proportion of surgically treated cases, increasing from 48.84% (2005–2008) to 64.1% (2009–2011), 72.4% (2012–2014), and 70.2% (2015–2017) (*p* < 0.001). The evolution of risk and protective factors for NEC trends over the study period are shown in Figure 2.

**Figure 3:**
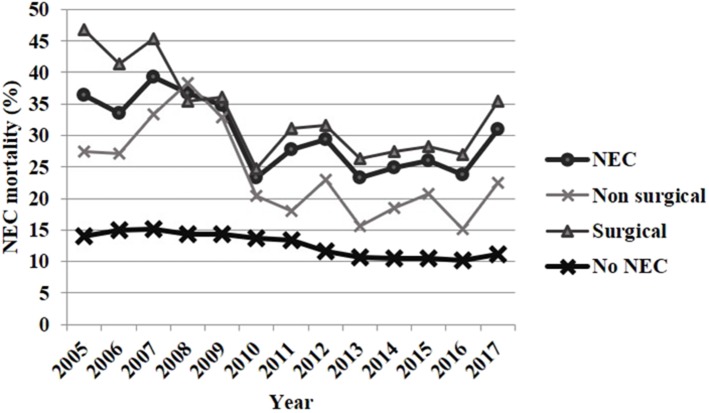
Evolution of necrotizing enterocolitis mortality by year from 2005 to 2017.

NEC mortality (Figure 3) decreased from 36.7% in the 2005–2008 period to 29.3% (2009–2011) and then to 26% (2012–2014) and 26.6% in the 2015–2017 period (*p* < 0.001). This significant decrease was seen in both subgroups, surgically, and non-surgically treated NEC patients. In the non-surgical subgroup, it changed from 31.7% (2005–2008) to 24.9% (2009–2011) and then to 19.1% (2012–2014) and 19.1% (2015–2017) (*p* = 0.004). In the surgically treated patients, mortality trended as follows: 41.9% (2005–2008) to 31.1% (2009–2011), 28.7% (2012–2014), and 29.8% (2015–2017) (*p* = 0.001). Also, in patients without NEC, mortality rate evolved over the study period, 14.6% (2005–2008), 13.8% (2009–2011), 10.9% (2012–2014), and 10.6% (2015–2017) (*p* < 0.001), whereas the mean age at death did not vary over these years: 17.5 ± 33.2 (2005–2008), 14.3 ± 23.9 (2009–2011), 17.5 ± 32.6 (2012–2014), and 16.7 ± 37.7 days (*p* = 0.09).

### NEC Associated Co-morbidity and Mortality

**Figure 4:**
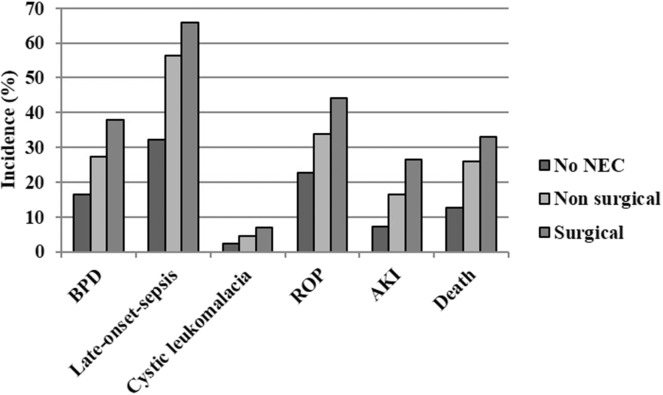
Co-morbidity and mortality associated with necrotizing enterocolitis classified depending on the treatment (only medical or surgical). AKI, acute kidney injury; BPD, bronchopulmonary dysplasia; ROP, retinopathy of prematurity.

There was a statistically significant association between NEC, some common preterm's diseases (BPD, late-onset sepsis, cystic leukomalacia, retinopathy of prematurity, acute kidney injury), and death as shown in Table 2. The risk increased significantly when comparing preterm infants with no NEC with non-surgically treated NEC and finally with surgically treated NEC as seen in Figure 4. Table 3 provides data about the incidence evolution from 2005 to 2017 of these NEC-associated co-morbidities depending on NEC diagnosis and reported treatment. In patients who had surgically treated NEC and died, age at death was 36.1 ± 34.3 (2005–2008), 36.7 ± 36.4 (2009–2011), 37.8 ± 42.3 (2012–2014), and 36.1 ± 33.6 days (2015–2017) (*p* = 0.98). Mean age at death among non-surviving NEC patients without surgical treatment trended as follows: 23.3 ± 27.7 (2005–2008), 24.1 ± 26.3 (2009–2011), 23.5 ± 26.6 (2012–2014), and 15.6 ± 10.6 days (2015–2017) (*p* = 0.54).

**Table 2:** Risk of morbi-mortality associated with medically and surgically treated necrotizing enterocolitis compared to preterm infants without necrotizing enterocolitis, adjusted by gestational age.

	**NEC**	**OR (95% CI)**
Bronchopulmonary dysplasia	Non-surgical	1.44 (1.18–1.77)
	Surgical	2.00 (1.71–2.33)
Late-onset sepsis	Non-surgical	2.66 (2.28–3.11)
	Surgical	3.22 (2.85–3.64)
Cystic periventricular leukomalacia	Non-surgical	1.70 (1.21–2.40)
	Surgical	2.50 (1.98–3.16)
Retinopathy of prematurity	Non-surgical	1.26 (1.04–1.51)
	Surgical	1.60 (1.38–1.85)
Acute kidney injury	Non-surgical	1.89 (1.54–2.32)
	Surgical	3.11 (2.70–3.58)
Death	Non-surgical	1.80 (1.51–2.14)
	Surgical	2.05 (1.79–2.34)

**Table 3:** Evolution of NEC associated co-morbidities of prematurity according to NEC diagnosis and treatment.

		**2005–2008**	**2009–2011**	**2012–2014**	**2015–2017**	***p***
BPD	No NEC	16.2 (799/4926)	16.3 (646/3955)	17.9 (765/4275)	15.7 (540/3443)	**0.048**
	Non-surgical	22.6 (51/226)	28.4 (36/127)	35.1 (40/114)	27.5 (25/91)	0.107
	Surgical	41 (87/212)	38.1 (91/239)	32.3 (83/257)	40.9 (85/208)	0.162
LONS	No NEC	34 (1940/5710)	33.6 (1609/4792)	31.9 (1558/4879)	28.9 (1233/4270)	**<0.001**
	Non-surgical	58.5 (179/306)	57.1 (101/177)	65.9 (81/123)	59 (69/117)	0.450
	Surgical	70.1 (223/318)	64.1 (209/326)	61.1 (218/357)	69 (207/300)	**0.047**
cPVL	No NEC	2.2 (159/7182)	2.7 (149/5487)	2.1 (119/5678)	2.3 (105/4606)	0.151
	Non-surgical	3.3 (12/367)	5.2 (10/193)	6.2 (9/145)	4.7 (6/127)	0.423
	Surgical	6 (21/349)	7.3 (25/343)	4.8 (18/374)	9.7 (29/300)	0.088
ROP	No NEC	25 (1368/5463)	21.8 (979/4482)	22.2 (1047/4725)	21 (836/3988)	**<0.001**
	Non-surgical	36.7 (91/248)	27.2 (40/147)	38.7 (48/124)	30.7 (31/101)	0.135
	Surgical	50 (102/204)	45.1 (116/257)	37.9 (105/277)	44.8 (100/223)	0.061
AKI	No NEC	7.8 (502/6436)	8.3 (435/5273)	6.5 (345/5299)	6.4 (297/4641)	**<0.001**
	Non-surgical	21.1 (70/332)	12.8 (24/187)	14.7 (19/129)	10.9 (14/128)	**0.020**
	Surgical	28.2 (86/305)	27.7 (92/332)	24.8 (90/363)	26 (79/304)	0.734

*Data are percentage (n/n of patients with valid data). p values are Fisher exact test (bold indicates statistical significance considered as p < 0.05). BPD, bronchopulmonary dysplasia as oxygen at 36 weeks postmenstrual age; LONS, late-onset sepsis; cPVL, cystic periventricular leukomalacia; ROP, retinopathy of prematurity; AKI, acute kidney injury; NEC, necrotizing enterocolitis*.

## Discussion

In our cohort, NEC incidence remained stable. According to the literature, an increased incidence has been reported in Sweden (1987–2009) and the Netherlands (2005–2013), whereas the incidence decreased in Australia (1986–1999) and Canada (2013–2017) and remained stable in Switzerland (2000–2012) and in the United States (1993–2012) (4, 11–15). The increased incidence in the Dutch and Swedish studies was attributed to the increased early survival rate and lower gestational age at birth over the study period. In our cohort, the mean gestational age at birth and age at death remained invariable over the study period, which could partly explain why the incidence did not increase. In the Swiss and American cohorts, the mean gestational age also remained unchanged. Of note, the incidence of NEC in our cohort remained mostly unchanged despite the higher rate of exclusive breast milk feeding at discharge and the implementation of probiotics during the study period. Overall, prophylactic probiotics have proved to be protective against NEC (16). However, this seems to be true only for some strains (17) and new studies should clarify which ones are more effective to reduce NEC. During the study period, probiotics were introduced but not universally. A recent survey among Spanish neonatal units showed that only in 23% are prophylactic probiotics used (18). Whether prophylactic probiotics failed in our cohort or the proportion of patients who received probiotics was insufficient remains to be clarified. Breast milk is a protective factor for NEC too. There is a dose response; the more human milk consumed, the less the risk (19). Donor milk is superior to formula but not to breast milk regarding NEC prevention (19). Donor milk, which is increasingly available in our units (87%) (18), has proved to facilitate exclusive breastfeeding at discharge (20). More data about the proportions of breast, donor, and formula milk received from birth to discharge per patient, which we lacked, would be useful to clarify why increasing rates of breastfeeding at discharge—the only nutrition data available in the database—were not associated with a decreased NEC incidence.

In our cohort, the proportion of cases who underwent surgical intervention increased over the studied years. In our opinion, this is more likely to be related to a change in surgical attitudes rather than to an increased rate of perforation among infants with NEC. The indication for surgical treatment varies depending on the surgeon. Only intestinal perforation seems to be accepted as an absolute indication for surgery (21). However, clinical deterioration in the absence of pneumoperitoneum is also a common indication for surgical treatment (22). Surgical cases have high mortality according to the literature (23). This highest mortality probably reflects the fact that the patients who underwent surgery were sicker. In our cohort, surgical cases had higher mortality than non-surgical cases too. However, mortality is also decreasing in the surgically treated NEC patients. Even more, the improvement in mortality is even greater than that seen in no surgical NEC cases and preterm infants without NEC. Decreased NEC mortality (all cases) over the years has also been reported by Ahle et al. in Sweden (4), whereas no changes in mortality over time were found by Heida et al. in the Netherlands (11) or Luig in Australia (12). Interestingly, Heida et al. reported a decreasing rate of surgical interventions (53 to 29%) from 2005 to 2013 in their Dutch cohort (11). They reported a significant decrease in peritoneal drainage insertion (28 to 12%) and a small increment in laparotomy indicated because of pneumoperitoneum (24 to 30%).

Finally, another finding of this study is that morbidity rates decreased over the study period in preterm infants without NEC, whereas this did not occur in infants with NEC diagnosis, which shows a niche for improvement. NEC leads to a systemic inflammatory state (24). Inflammation plays an essential role in the pathophysiology of several complications associated with prematurity like bronchopulmonary dysplasia, retinopathy of prematurity, and cystic periventricular leukomalacia. In fact, NEC is known to be associated with an increased risk of having these complications (25). Current NEC treatments do not focus on reducing this inflammatory state directly. Surgery aims to remove gangrenous bowel segments, with the objective of reducing bacterial translocation, sepsis, and multiorgan dysfunction (23). Some authors have suggested that early laparotomy could improve the outcomes, but the optimal indications and timing for surgery in patients with NEC beyond perforation remain to be clarified (26).

The main limitation of this study is the lack of data regarding the reason for surgical indication, surgical procedures, and timings in the original database. Data about bowel-related NEC complications (i.e., intestinal post-NEC necrosis, short bowel syndrome) are also not collected and therefore could not be studied. Finally, important nutritional data (i.e., proportions of the different feedings) were not available either. A recent survey published by our group describes the current practices regarding NEC in Spain. This survey showed great variation regarding surgical indications and preferred interventions (18). Among the surveyed surgeons, 19% reported that the indications for surgery have remained the same, and 41% said that surgical indications have become more conservative over the last 10 years. On the contrary, 37% considered that early interventions, even in the absence of perforation, were becoming more frequent. However, these surgeons usually worked in high volume centers. The multicenter nature, the prospective data collection, and the big sample size are strengths of the present study. This has allowed us to study the relationship between NEC and other common complications of the preterm infant, for which NEC seems to be a risk factor. This aspect has seldom been described using large cohorts datasets.

Necrotizing enterocolitis incidence remained unchanged over the study period; thus, implementation and search for new preventive strategies are still required. Interestingly, the proportion of NEC patients who underwent surgery trended up from 2005 to 2017. Mortality improved during the studied years in preterm infants with and without NEC diagnosis. However, it has improved more in patients with NEC than in preterm infants without NEC. On the other hand, the incidence of common co-morbidities of preterm birth, in general, improved in patients without NEC while it remained mostly invariable in patients with NEC. New treatments should focus on reducing the common preterm infant co-morbidities in patients with NEC, probably targeting the systemic inflammatory reaction associated with NEC.

## Data Availability Statement

The datasets generated for this study are available on request to the corresponding author.

## Ethics Statement

Local Ethics Research Committee of all participant centres approved the data collection protocol when they joined the network. Permission for data analysis was obtained from the executive committee of the Spanish neonatal SEN1500 network. Written informed consent from the participants' legal guardian/next of kin was not required to participate in this study in accordance with the national legislation and the institutional requirements.

## Author Contributions

All authors contributed in the study design. CZ performed the statistical analysis and drafted the initial manuscript along with IG. AA-A, NO, TS, ES, MS, FG-MR, and MC contributed to interpreting the results and revised the manuscript making important intellectual contributions.

## Conflict of Interest

The authors declare that the research was conducted in the absence of any commercial or financial relationships that could be construed as a potential conflict of interest.
